# Identification of novel off targets of baricitinib and tofacitinib by machine learning with a focus on thrombosis and viral infection

**DOI:** 10.1038/s41598-022-11879-1

**Published:** 2022-05-12

**Authors:** Maria L. Faquetti, Francesca Grisoni, Petra Schneider, Gisbert Schneider, Andrea M. Burden

**Affiliations:** 1 Department of Chemistry and Applied Biosciences, Institute of Pharmaceutical Sciences, ETH Zurich, Zurich, Switzerland; 2 Department of Biomedical Engineering, Institute for Complex Molecular Sciences, Eindhoven University of Technology, Eindhoven, The Netherlands; 3 inSili.com LLC, Zurich, Switzerland; 4 ETH Singapore SEC Ltd, Singapore, 138602 Singapore

**Keywords:** Rheumatic diseases, Target identification, Target validation

## Abstract

As there are no clear on-target mechanisms that explain the increased risk for thrombosis and viral infection or reactivation associated with JAK inhibitors, the observed elevated risk may be a result of an off-target effect. Computational approaches combined with in vitro studies can be used to predict and validate the potential for an approved drug to interact with additional (often unwanted) targets and identify potential safety-related concerns. Potential off-targets of the JAK inhibitors baricitinib and tofacitinib were identified using two established machine learning approaches based on ligand similarity. The identified targets related to thrombosis or viral infection/reactivation were subsequently validated using in vitro assays. Inhibitory activity was identified for four drug-target pairs (PDE10A [baricitinib], TRPM6 [tofacitinib], PKN2 [baricitinib, tofacitinib]). Previously unknown off-target interactions of the two JAK inhibitors were identified. As the proposed pharmacological effects of these interactions include attenuation of pulmonary vascular remodeling, modulation of HCV response, and hypomagnesemia, the newly identified off-target interactions cannot explain an increased risk of thrombosis or viral infection/reactivation. While further evidence is required to explain both the elevated thrombosis and viral infection/reactivation risk, our results add to the evidence that these JAK inhibitors are promiscuous binders and highlight the potential for repurposing.

## Introduction

**Figure 1:**
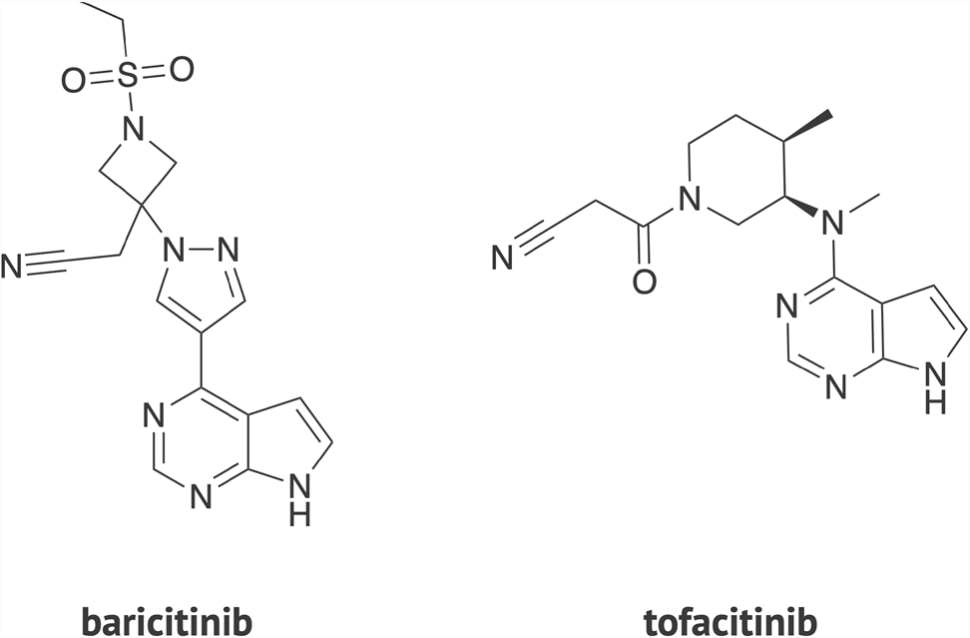
Chemical structure of baricitinib and tofacitinib. The two drugs were the first JAK inhibitors to receive approval in the USA and Europe to treat rheumatoid arthritis.

Janus Kinase (JAK) (EC number 2.7.10.2) inhibitors are novel targeted synthetic disease-modifying antirheumatic drugs. The new class of small molecule drugs represents an important alternative to treat moderate-to-severe rheumatoid arthritis (RA) patients with non- or inadequate response to conventional synthetic disease-modifying antirheumatic drugs (sDMARD) and biological disease-modifying antirheumatic drugs (bDMARD)^1^*.* The JAK inhibitors target one or more kinases of the JAK family (JAK1, JAK2, JAK3, and non-receptor tyrosine-protein kinase TYK2) and inhibit multiple pro-inflammatory cytokines critical to the pathogenesis of autoimmunity, such as interleukin (IL)-6, IL-10, and interferon (IFN)-γ^2–4^. Baricitinib (JAK1/JAK2 inhibitor) and tofacitinib (JAK1/JAK3 inhibitor) are the first members of this class approved in the United States (US) and Europe to treat RA (Fig. 1).

Safety concerns associated with JAK inhibitors, such as the increased risk for thrombosis and viral infection or reactivation, have emerged worldwide, and boxed warnings are included on all approved JAK inhibitors used to treat inflammatory conditions^5–11^. While a dose–response effect was observed in the risk of thrombosis in clinical trials of both baricitinib and tofacitinib, there is no known mechanism associated with the pharmacological on-target effect that could explain the risk of thrombosis associated with baricitinib and tofacitinib. Although the use of baricitinib and tofacitinib is expected to increase infections due to modulation of IFNs^12^, the incidence of Herpes Zoster (HZ), particularly associated with JAK inhibitors drugs, remain unclear^13,14^. Thus, the increased risk of these safety concerns is heavily debated.

It is well established that unintended off-target activity may interfere in multiple biological processes, inducing undesired side effects^15^. Nevertheless, given the complexity of the human proteome, complete elucidation of all biological targets of a drug before its entrance into the market is often unfeasible. In this context, machine learning can be used to predict the potential for an approved drug to interact with off-targets and identify potential safety-related concerns^16^. The identification of additional drug-target interactions using chemo-centric and machine learning approaches and experimental confirmation may help to determine mechanisms of adverse drug events^17,18^. For example, previously unknown drug-target interactions for the approved compound Celecoxib were identified using a ligand-based method, which is based on the principle that structural similarity reflects functional similarity, supporting the biological plausibility of reported cardiovascular adverse drug events^19,20^. Moreover, off-target profiling is frequently used to identify candidate drugs for repurposing. For example, computational studies using machine learning identified baricitinib as a promising JAK inhibitor for repurposing in patients with severe acute respiratory syndrome coronavirus 2 (SARS-CoV-2 or COVID-19)^21,22^. Baricitinib was considered a potential candidate for repurposing in COVID-19 based on the high affinity for AP-2 associated protein kinase 1 (AAK1) (EC number 2.7.11.1), which is critical in regulating viral endocytosis, and its inhibition may reduce the ability of the virus to infect lung cells^23^.

Improving our understanding of the target space of JAK inhibitors drugs is essential in order to explain the mechanisms of unexpected side-effects associated with these drugs and to identify opportunities for repurposing. Although several binding screens of tofacitinib and baricitinib have been published, they are mostly limited to a few protein families, such as protein kinases and lipid kinases^24–26^. Thus, the community would benefit from a more extensive characterization of the target profile of tofacitinib and baricitinib.

In light of the currently unexplained thrombotic and viral infection risk and the previously observed off-target binding potential of baricitinib, here we investigate if the thrombosis and viral infection/reactivation risk may be a result of an off-target effect. We, therefore, aimed to leverage well-established machine learning methods to identify off-target drug-protein interactions for baricitinib and tofacitinib and validate such predictions in vitro assays. Previously unknown off-targets of baricitinib and tofacitinib were predicted and confirmed drug-target interactions suggest an attenuation of pulmonary vascular remodeling, modulation of Hepatitis C (HCV) viral response, and hypomagnesemia. Nevertheless, the identified off-target interactions could not explain the elevated thrombosis or viral infections/reactivation risk. These results suggest both JAK inhibitors as potential candidates for drug repurposing.

## Results

### Off-target profiling of baricitinib and tofacitinib by machine learning revealed additional drug-target interactions

Macromolecular targets of baricitinib and tofacitinib were predicted using two previously published machine learning approaches: Target Inference Generator (TIGER)^20^ and SOM-based Prediction of Drug Equivalence Relationships (SPiDER)^27^. Both approaches follow the chemical similarity principle, in which molecules sharing similar structures are likely to have similar bioactivity^28^.

SPiDER uses a neural network (Self-organizing map [SOM]), and drug–target relationships are inferred based on descriptor similarity of a query compound to reference ligands without directly considering the target similarity. The method uses topological and physicochemical information of molecules to suggest a functional similarity between compounds. TIGER extends SPiDER using a more extensive set of targets, as well as a different prediction algorithm and scoring function. Both approaches have been extensively applied to de novo designed compounds, natural products with biological activity, and approved drugs^29–31^.

Overall, 40 potential targets for baricitinib and 58 for tofacitinib (SPiDER [p < 0.05] and/or TIGER [score > 1]) were predicted by TIGER and SPiDER. The list of predicted targets accompanied by the score for TIGER, and p-values for SPiDER, are shown in Supplementary Tables S1 and S2, respectively. The cutoff values were chosen based on recent prospective studies where the target prediction tools led to bioactivity confirmation in vitro^20,32,33^. The resulting predictions reflect both known and unknown potential drug-target interactions. From all predicted targets, nine targets for baricitinib and eight for tofacitinib were identified as being relevant for thrombosis and viral infection/reactivation (Table 1).

**Table 1:** Suggested targets with impact on thrombosis and viral infection per JAK inhibitor drug and target prediction approach.

Drug	Predicted target	Approach		Thrombosis	Viral infection/reactivation
		TIGER	SPiDER		
Baricitinib	Protein Kinase C Beta (PKC-β)	x		x	
	Adenosine Receptor A2A (AA2AR)	x		x	
	Inducible Nitric Oxide Synthase (iNOS)	x		x	
	Phosphodiesterase 10A (PDE10A)	x	x	x	
	Ras Related Protein Rab-7a	x		x	
	Epidermal growth factor receptor (EGFR) kinase	x			x
	Deoxycytidine kinase (DCK)	x			x
	Serine/threonine-protein kinase N2 (PKN2)^a^		x		x
	Thymidine kinase (HSV)^b^	x			x
Tofacitinib	Arachidonate 15-Lipoxygenase (15-ALOX)	x		x	
	Adenosine Receptor A2A (AA2AR)	x		x	
	Short transient receptor potential channel 6 (TRPC6)	x		x	
	Short transient receptor potential channel 3 (TRPC3)	x		x	
	Adenosine Receptor A3 (ADORA3)	x		x	
	Exportin-1 (XPO1)	x			x
	Serine/threonine-protein kinase N2 (PKN2)	x	x		x
	Ubiquitin-conjugating enzyme E2 N (Ubc13)	x			x

Commercial assays were unavailable for TRPC6 or TRPC3, and therefore, these targets could not be validated. Instead, transient receptor potential cation channel subfamily M member 6 (TRPM6) was employed for the respective binding assays.

^a^PKN2 was included in the list of targets tested for baricitinib, which allowed us to make a direct comparison between tofacitinib and baricitinib inhibitory activity on this target.

^b^Human herpesvirus 1 (strain SC16).

### In vitro characterization confirmed previously unknown baricitinib and tofacitinib drug-target interactions

Of the 98 predicted, a total of 11 drug-target interactions were experimentally validated using biochemical or cell-based assays, based on the availability of fee-based in vitro testing services (Table 2). Among the predicted targets, two members of the Transient Receptor Potential superfamily of calcium channels were suggested, namely short transient receptor potential channels 6 (TRPC6) and 3 (TRPC3). Commercial assays were unavailable for TRPC6 or TRPC3, and therefore, these targets could not be tested. Instead, transient receptor potential cation channel subfamily M member 6 (TRPM6) (EC number 2.7.11.1) was employed for the respective binding assays. Additionally, while serine/threonine-protein kinase N2 (PKN2) (EC number 2.7.11.13) was among the predicted targets for tofacitinib, but not for baricitinib, PKN2-baricitinib binding affinity was previously determined in baricitinib (apparent dissociation constant [*K*_d app_] = 269 nM and IC_50_ = 284 nM)^25^. Thus, PKN2 was included in the list of targets tested for baricitinib, allowing a direct comparison between tofacitinib and baricitinib inhibitory activity on this target in the same experimental conditions.

**Table 2:** In vitro findings for baricitinib and tofacitinib off-target activity.

Drug	Safety issue	Target	IC50 (μM)^a^	*K*_i_ or *K*_d_ (μM)
Baricitinib	Thrombosis	Adenosine Receptor A2A (AA2AR)^b^	Inactive	n.d
		Inducible NOS (iNOS)	Inactive	n.d
		PI3 Kinase (p110b/p85a)	Inactive	n.d
		Phosphodiesterase 10A2 (PDE10A2)	28 ± 2^d,e^	*K*_i_ = 6.1
	Viral infection	Serine/threonine-protein kinase N2 (PKN2)	0.24, 0.21^e^	*K*_i_ = 0.082, 0.069^e^
		Epidermal growth factor receptor (EGFR)	Inactive	n.d
Tofacitinib	Thrombosis	Adenosine Receptor A3 (ADORA3)^b,c^	Inactive	n.d
		Arachidonate 15-lipoxygenase (15-ALOX)	Inactive	n.d
		Transient receptor potential cation channel subfamily M member 6 (TRPM6)^f^	n.d	*K*_*d*_ = 6.1, 7.7^e^
		Adenosine Receptor A2A (AA2AR)^c^	Inactive	n.d
	Viral infection	Serine/threonine-protein kinase N2 (PKN2)	0.71, 0.74^e^	*K*_i_ = 0.24, 0.25^e^

All in vitro testing was performed on a fee-for-service basis at Eurofins Cerep (www.eurofins.com).

*n.d:* not determined.

^a^JAK inhibitors were tested at a concentration of 30 μM. During follow-up experiments, JAK inhibitors were tested in multiple concentrations (top concentration of 100 μM) for dose–response curve characterization and determination IC_50_/EC_50_ (two or three replicates).

^b^Antagonistic effect.

^c^Agonistic effect.

^d^Values are the mean ± standard error of the mean (SEM) for the number of replicates (*n*) > 2.

^e^For *n* = 2, no averaging was made, and both values are presented.

^f^Commercial assays were unavailable for TRPC6 or TRPC3, and therefore, these targets could not be validated. Instead, transient receptor potential cation channel subfamily M member 6 (TRPM6) was employed for the respective binding assays.

From the 11 drug-target interactions tested, five showed an experimental readout greater than 25% drug-target interaction at 30 μM and were selected for further in vitro characterization (Table 2). Four out of five drug-target interactions were confirmed by further in vitro evaluation, with IC_50_ and *K*_i_ or *K*_d_ values in the nanomolar range (baricitinib and tofacitinib on PKN2) and in the micromolar range (baricitinib on Phosphodiesterase 10A2 (PDE10A2) EC number 3.1.4.17; tofacitinib on TRPM6). The investigated drugs were considered as active if the determined IC_50_ was lower than 30 μM. The raw in vitro data for drug-binding activity using biochemical assays is available in the Supplementary Table S3. Dose–response curves for targets showing activity are available in the Supplementary Figs. S1–S4.

### Computational ligand docking predicted potential modes of baricitinib and tofacitinib-target interaction

**Figure 2:**
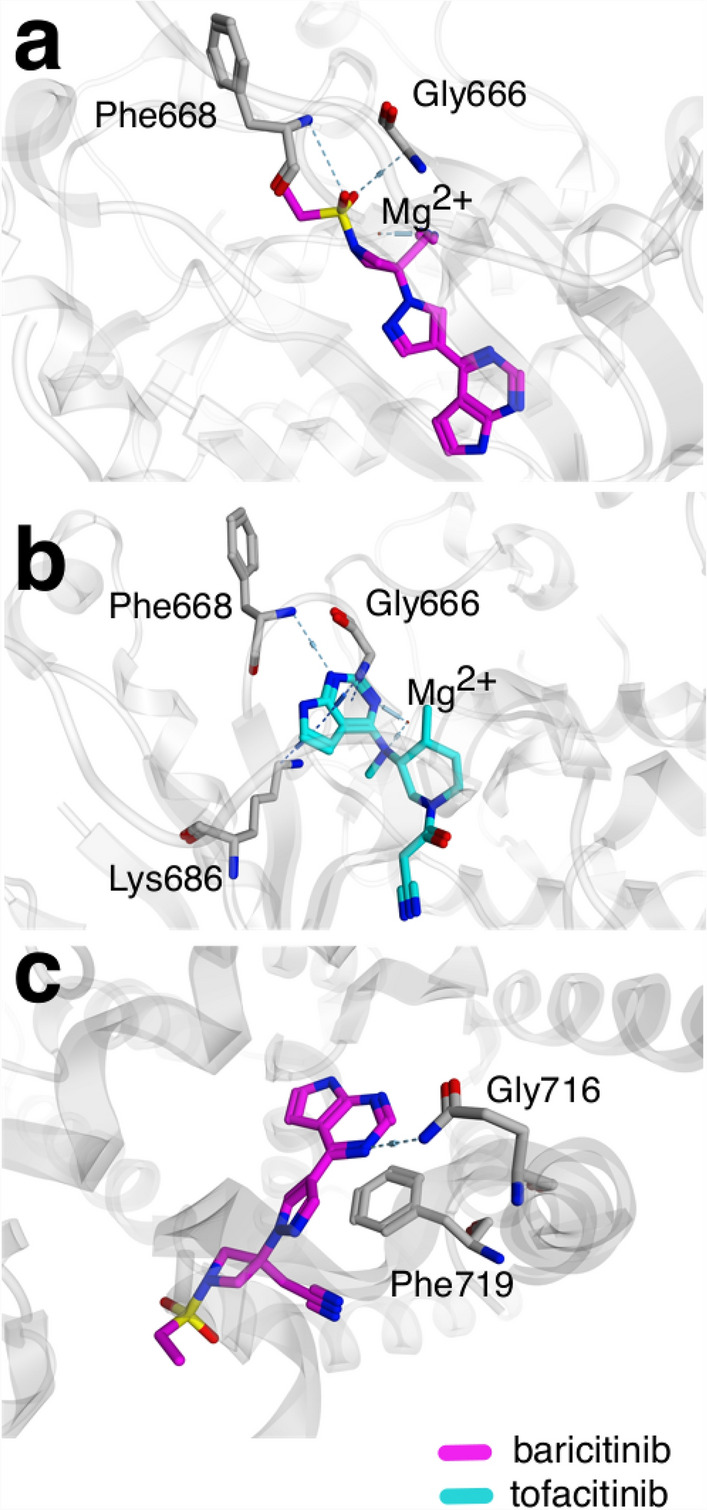
Predicted docking pose of baricitinib and tofacitinib on the identified targets. Predicted docking pose of baricitinib (**a**) and tofacitinib (**b**) in the binding site of PKN2 (PDB-ID: 4CRS^34^). Predicted binding pose of baricitinib (**c**) in the binding site of PDE10A (PDB-ID: 5C28^35^).

Computational ligand docking (Fig. 2) predicted potential modes of interaction (i.e., three-dimensional orientations of the drug molecule and a target) for baricitinib and tofacitinib in the binding pocket of the identified macromolecular targets (PKN2 [PDB-ID: 4CRS^34^]; PDE10A [PDB-ID: 5C28^35^]) using docking algorithms, and it provided the respective score for each orientation predicted. TRPM6 was not considered due to the unavailability of an experimentally determined structure.

Molecular docking of baricitinib to PKN2, shown in Fig. 2a, suggests an interaction between the nitrile nitrogen on the drug structure and Mg^2+^. Similar to the crystallized ligand, the two residues Phee668 and Gly666, are hydrogen donors and interact with one oxygen from the sulfonyl group. Additionally, two arene-H interactions between pyrrole on the tofacitinib structure and the hydrogen on the amino groups of PKN2 residue Gly666 were suggested in Fig. 2b. A third arene-H interaction between pyrimidine on the tofacitinib molecule and the hydrogen on the amino groups of Lys686 was indicated. Like the crystallized ligand, an interaction with Phe668 is predicted. Additionally, interactions between Mg^2+^ and two nitrogen atoms are suggested.

In Fig. 2c, the Gln716 is making a hydrogen bond, donating a bond to the core pyrimidine nitrogen. Another interaction is suggested between the pyrimidine core of baricitinib and the protein—a π-stacking interaction with the key residue Phe719. Some structural equivalence between baricitinib and the co-crystallized ligand is observed, such as the pyrimidine core in the earliest and the aromatic ring in the latest. The interaction with Gln716 and Phe716 are key for recognition of PDE10A inhibitors by the enzyme^36^.

## Discussion

Both baricitinib and tofacitinib were confirmed as “promiscuous drugs” since they bind to proteins from families other than the primary therapeutic target^20^. Thus, both drugs may be potential candidates for adverse drug effects and further repurposing. The confirmed drug-target interactions suggest an attenuation of pulmonary vascular remodeling (inhibition of PDE10A), modulation of Hepatitis C (HCV) viral response (inhibition of PKN2), and hypomagnesemia (inhibition of TRPM6). Therefore, we did not identify off-target interactions that could explain the elevated thrombosis or viral infections/reactivation risk observed in the clinical setting^5,37,38^.

The thrombotic and cardiovascular risk associated with JAK inhibitors remains debated, which is largely due to a lack of a clear mechanism associated with the therapeutic target that could explain the increased risk. In our analysis, we aimed to investigate if there may be a plausible off-target interaction that could explain the observed effects. While the computational approaches identified several targets relevant for blood coagulation and platelet aggregation (e.g., Adenosine receptor A2A [AA2AR] and Arachidonate 15-lipoxygenase [15-ALOX] EC number 1.13.11.33), neither baricitinib nor tofacitinib was found to interact with those receptors in vitro, ruling them out as potential off-targets.

Nonetheless, the drugs were shown to inhibit two targets related to thrombosis—PDE10A and TRPM6. PDE10A, which was recently validated as a novel target to treat pulmonary arterial hypertension (PAH) due to its central role in progressive pulmonary vascular remodeling^39,40^, was identified as a target of baricitinib. The preliminary in vitro results of this study showed moderate inhibition of baricitinib for PDE10A2. Molecular docking in the active site of PDE10A (Fig. 2c) suggested a similar binding pose of baricitinib to the crystallized inhibitor (PDB ID: 5C28), with a predicted π-stacking interaction with the Phe719 residue, crucial for biological activity^36^. Additionally, among important regions for ligand binding is the occupation of a hydrophobic clamp formed by two phenylalanine residues, Phe719 and Phe686. The arene-H-type interaction between the pyrazole structure in baricitinib and Phe686 residue while occupying the hydrophobic clamp suggests that baricitinib has a similar binding mode to PQ-10, a papaverine analog having IC_50_ equal to 6 μM.

Clinically, PDE10A inhibition is expected to decrease the risk for thrombosis, particularly in patients with PAH. Thus, the expected positive clinical impact of PDE10A inhibition on the risk of thrombosis is not in line with a potential link to an elevated thrombosis risk. Rather, baricitinib might improve progressive pulmonary vascular remodeling.

This study further identified previously unknown off-target interactions of tofacitinib with TRPM6, with moderate binding affinity. While our computational approach identified TRPC6 and TRPC3 as potential targets, we were unable to experimentally validate these targets due to a lack of commercially available in vitro assays. Thus, we can only speculate that the binding affinity observed with TRPM6 may translate to binding in TRPC6 and TRPC3. Additional experiments are needed to confirm if the C subfamily is also a potential target of tofacitinib. This aspect is important as TRPC6 is known to regulate human clot retraction, physiological hemostasis, and thrombus formation, and its inhibition is thought to have a positive effect on thrombotic outcomes^43^. Thus, further research is needed to confirm if tofacitinib binds to TRPC6.

Cumulatively, the active targets in this study suggest that JAK inhibitors may have a beneficial effect on cardiovascular risk, and therefore do not support a hypothesis that the risk of thrombosis is related to an off-target drug effect (in the framework of the macromolecular targets investigated in this study). Nevertheless, we note that recent US-based cohort studies that have identified no difference in thrombosis risk between tofacitinib and TNF-inhibitors^44–46^, thereby suggesting that much of the observed risk seen in pharmacovigilance studies^6,47^ may be due to underlying risk factors rather than a drug effect. For example, standardized incidence rates (IR) of venous thromboembolism or pulmonary embolism were comparable among patients with rheumatoid arthritis using tofacitinib (IR = 1.05 [0.78–1.39]) and bDMARDs (IR = 0.94 [0.85–1.03]) within MarketScan database cohorts^46^. Conversely, recent analysis using pharmacovigilance data of the US FDA Adverse Event Report System (FAERS) did not identify a signal of disproportionate reporting for venous thromboembolism and/or pulmonary embolism events with tofacitinib^46^. Therefore, an improved understanding of the underlying risk factors for thrombosis in patients with JAK inhibitors is urgently needed.

The risk of thrombosis can be further increased in rheumatoid arthritis patients with high disease activity, cardiovascular risk factors (e.g., obesity), immobility, and hormonal replacement therapy^48,49^. Patients using JAK inhibitors frequently have high disease activity with non- or inadequate response to csDMARDs and bDMARDs and multiple chronic conditions (e.g., cardiovascular disorders and depression), which can make the attribution of thrombotic events in patients treated with JAK inhibitors even more intricate.

In addition to thrombosis, targets related to viral infection and viral reactivation were investigated. Therapies targeting the JAK family of enzymes may interfere with a normal antiviral response, including inhibition of IFN-γ activity, and may potentially increase the risk of infection/ reactivation of several viral infectious diseases, particularly HZ in rheumatoid arthritis patients^14,38^. The computational approaches identified several targets expected to play a role in viral endocytosis and viral response, including epidermal growth factor receptor (EGFR) kinase (EC number 2.7.10.1) and PKN2. Although baricitinib and tofacitinib were not found to interact with EGFR in our experimental setting used, both showed PKN2 inhibitory activity.

Clinically, PKN2 is of great importance as a target for antiviral therapy, particularly anti-HCV, as its suppression leads to viral replication blockage in humans. PKN2 inhibitors, in combination with other antiviral therapies, have demonstrated synergistic antiviral activity for chronic HCV treatment^50,51^. While, to date, three studies have evaluated tofacitinib binding activity on PKN2, the results are contradictory^24,25,52^.

The preliminary in vitro results of the current study suggest PKN2 inhibition with both baricitinib and tofacitinib, as the IC_50_ and *K*_i_ values are in the nanomolar range. Moreover, the molecular docking in the PKN2 crystalized structure suggested a similar binding mode, shape, and certain molecular features (i.e., pharmacophore) of baricitinib and tofacitinib (Fig. 1) as to the co-crystalized ligand at the protein binding site. The model indicates that the two drugs interact with the Mg^2+^ similarly to the crystallized ligand—a PKN2 inhibitor—on the kinase functional pocket^53^. Similar to the crystallized ligand, each Phe668 and Gly666 as hydrogen donors and interact with one oxygen from the sulfonyl group in the baricitinib molecule, suggesting the role of this group for drug anchoring in the binding pocket. Additionally, the Phe668 residue backbone interacts with one nitrogen from the pyrimidine in tofacitinib, suggesting a similar binding mode to the crystallized ligand on the active pocket of PKN2. However, the impact of PKN2 inhibition is proposed to have a positive effect on viral suppression^51^, and therefore does not explain the elevated risk of HZ in rheumatoid arthritis patients. The exact mechanism of HZ viral reactivation remains unclear.

Outside of its role in viral suppression, PKN2 may play an essential role in various cellular processes, such as cellular proliferation, migration, and signaling pathways^54–56^. Moreover, PKN2 is involved in autoinflammatory disorders^57^, heart failure^58^, and it is a target of interest in cancer^56,59,60^. As concerns regarding the risk of malignancy and major adverse cardiovascular events (MACE) in patients treated with tofacitinib have been raised by the European Medicines Agency, it is important to consider the potential role of PKN2 inhibition^61^, However, in mice models, PKN2 activation was the cause of cardiac dysfunctions^58^, and therefore, the clinical impact of PKN2 inhibition is contradictory to the risk of cancer and MACE in rheumatoid arthritis patients.

Off-target profiling using computational approaches has been widely used to identify candidates for drug repurposing^62,63^. Indeed, JAK inhibitors were recently established as potential candidate therapies for COVID-19 based on in silico methods^64–66^. Our computational methods identified 98 drug-target predictions, and the preliminary in vitro results found inhibitory activity on several proteins other than the primary therapeutic target, thereby confirming baricitinib and tofacitinib as promiscuous drugs and candidates for drug repurposing studies. For example, PDE10A inhibition has been primarily studied in psychiatric and neurological conditions, such as schizophrenia^67^ and Huntington’s disease^68^, and, to a lesser extent, in multiple peripheral pathological conditions^69,70^ (e.g., osteogenic differentiation). Additionally, PDE10A inhibition by baricitinib is hypothesized to have a synergistic pharmacological effect in combination with other COVID-19 treatments (e.g., antiviral and corticosteroids drugs) due to the anti-fibrotic and anti-inflammatory effects of phosphodiesterase’s inhibitors on the treatment of COVID-19 and its associated conditions (e.g., thrombosis, inflammation, and fibrosis)^71,72^. Therefore, the confirmed PDE10A inhibition identified in this study supports the potential for baricitinib as a potential candidate outside of rheumatology.

Moreover, while TRPM6 was not initially predicted, the moderate inhibitory activity is worth investigating. TRPM6 inhibition is not fully elucidated. However, it is mainly involved in magnesium homeostasis in the intestine and kidney^73,74^*,* and it has been demonstrated to have expression levels modulated by hormones such as estrogen^75^ and angiotensin II^76^, immunosuppressant^77^, and diuretics drugs^78^, and epidermal growth factor (EGF)^79^. Moreover, the decreased expression of TRPM6 in cancer patients treated with EGFR targeted therapies (e.g., cetuximab) seems to positively contribute to the oncologic efficacy of these therapies, as decreased magnesium availability inhibits cell proliferation and slows down tumor growth^79,80^. Thus, we encourage further investigation into the clinical relevance of TRPM6 inhibition by tofacitinib in oncology.

The results also highlight the complementarity of the two approaches TIGER and SPiDER. JAK3 (TIGER score = 6.9) and JAK1 (TIGER score = 1.9) ranked fourth and twenty-sixty, respectively, on the list of predicted target proteins for tofacitinib, while JAK3 (TIGER score = 8.4) ranked second for baricitinib using the TIGER approach. Tyrosine Kinase (EC number 2.7.10.1) (tofacitinib [p-value = 0.01]; baricitinib [p-value = 0.02]) ranked second on the list for the two JAK inhibitors using SPiDER. Although lacking the subfamily specificity, SPiDER correctly identified the target family, which encompasses the JAK kinases.

The top predictions for tofacitinib suggested D-Amino-Acid Oxidase (EC number 1.4.3.3; TIGER score = 11.3), and Phosphodiesterase (3',5'-Cyclic-Nucleotide Phosphodiesterase; EC number 3.1.4.17; SPiDER p-value = 0.009), while the top predictions for baricitinib pointed to Deoxycytidine Kinase (EC number 2.7.1.74; TIGER score = 8.8), and Monoamine Oxidase (EC number 1.4.3.4; SPiDER p-value = 0.016). Experimental validation of the remaining top-ranking predicted targets, including Deoxycytidine Kinase suggested as a new target of baricitinib, will be considered for future study.

Only a small fraction (~ 10%) of the 98 predicted off-targets were experimental tested in this study. However, as we did observe active binding on three distinct targets, this study suggests that there might be other interactions among the list of predicted targets. Thus, further testing might help to elucidate the molecular mechanisms of these JAK inhibitors and open the door for improved understanding of the safety concerns and repurposing in other conditions (e.g., in neurodegenerative diseases, diabetes, and viral infections).

The use of computational and experimental approaches in this study allowed for the identification and characterization of previously unknown off-target interactions for the two JAK inhibitors (e.g., baricitinib-PDE10A and tofacitinib-TRPM6), which adds to the target space of tofacitinib and baricitinib. TIGER and SPiDER identified additional targets of tofacitinib and baricitinib that other approaches, such as the Similarity Ensemble Approach (SEA)^81^ and the SwissTargetPrediction^82^, were unable to capture (Supplementary Table S4). For example, both SEA and SwissTargetPrediction failed to assign TRPM6 (tofacitinib) and PKN2 (baricitinib) identified by TIGER and SPiDER, respectively. Nevertheless, comparisons between different target prediction approaches should to be performed with caution, as extensive experimental studies are essential for validating the hypotheses and demonstrating the potential impact of each approach.

Moreover, TIGER and SPiDER use a large set of targets, encompassing a broad scope of protein families that allows identifying drug promiscuity. Additionally, the inclusion of multiple protein families helps to predict a broad off-target drug profile and point out potential targets for repurposing studies. This is particularly important for understudied druggable proteins and targets with no approved drugs. Ultimately, it increases knowledge on the potential drug effects of tofacitinib and baricitinib.

Despite the encouraging results of our study, we are mindful of some limitations. As identified, we could not experimentally validate all predicted targets related to thrombosis (e.g., TRPC6) or viral infection/reactivation (e.g., deoxycytidine kinase [DCK; EC number 2.7.1.74], Thymidine kinase [HSV; EC number 2.7.1.21], Exportin-1 [XPO1], or Ubiquitin-conjugating enzyme E2 N [Ube2N; EC number 2.3.2.23]). As such, we cannot conclude if these targets may play an important role in thrombosis or viral infection/reactivation risk and are limited in the conclusions we can draw. Thus, we encourage researchers with access to the appropriate assays to validate these targets. Moreover, there might be additional targets of relevance that were not predicted by our computational tools.

The provided docking poses constitute an additional support to the experimentally determined values and that shall not be considered as a binding hypothesis. Thus, future computational studies including X-ray crystallography analysis are needed, and it may provide insights on the binding mode of the JAK inhibitors on the new targets. We also acknowledge that the activity of small molecule drugs using in vitro assays does not always translate into activity in the cellular environment. Thus, the results should still be interpreted with caution and treated as preliminary evidence for the off-target binding of baricitinib and tofacitinib.

In summary, previously unknown off-targets of baricitinib and tofacitinib were identified and characterized using a combination of machine learning and experimental methods. The confirmed target interactions suggest an attenuation of pulmonary vascular remodeling, modulation of HCV viral response, and hypomagnesemia. Thus, it does not endorse the hypothesis of elevated thrombosis or viral infections/reactivation risk explained by one (or more) drug-target interactions. Consequently, the current safety concerns may be due to underlying patient-specific factors (confounders) or to targets not detected by our computational pipeline. Additionally, as not all of the predicted targets were experimentally validated, further research is warranted. Finally, baricitinib and tofacitinib may be potential candidates for repurposing, as they were identified as drugs with promiscuous binding activity.

## Methods

### Data preparation and molecular representation for target prediction

Baricitinib and tofacitinib were provided as Simplified Molecular Input Line Entry Specification (SMILES) and processed in KNIME v3.7.2^83^ with the MOE v.2019.0102^84^ “wash” function employing the following options: “disconnect salts”, “remove lone pairs”, “deprotonate strong acids”, “remove minor component”, “protonate strong bases,” and “add hydrogen”. Chemically advanced template search version 2 (CATS2)^85^ descriptors and two-dimensional MOE descriptors (‘QSAR descriptors’ node of KNIME; ’Forcefield’ = MMFF94*) were calculated for all generated molecules and used as input for the target prediction tools;

### Macromolecular target prediction and selection

Target Inference Generator ([TIGER v. 19.07], inSili.com. LLC, Zurich)^20^ and Self-organizing map–based prediction of drug equivalence relationships (SPiDER)^27^ software’s were used for target activity prediction. Targets with statistically meaningful predictions from SPiDER (p < 0.05) and/or TIGER (score > 1) were selected for in vitro characterization if they were considered to have a potential influence on thrombosis or viral infection/reactivation.

### In vitro characterization

Baricitinib (99.97% purity) and tofacitinib (99.96% purity) compounds were purchased from MedChemExpress LLC (New Jersey, www.medchemexpress.com). In vitro characterization was performed on a fee-for-service basis at Eurofins (www.eurofins.com) if the assay was commercially available.

For the biochemical assays, compound targets showing an experimental readout greater than 25% (inhibition or stimulation) at 30 µM were selected for follow-up, and dose–response curve characterization and determination IC_50_/EC_50_ (two or three replicates, multiple concentrations, maximum 100 µM concentration). Additional details on the conduct of in vitro assays are included in Supplementary Information S1.

### Computational ligand docking

Protein crystal structures of Serine/threonine-protein kinase N2 (PKN2) (PDB ID: 4CRS^34^) and Phosphodiesterase 10A (PDE10A) (PDB ID: 5C28^35^), were retrieved from the worldwide Protein Data Bank (wwPDB, https://www.rcsb.org/) and prepared for docking using MOE software (v.2019.0102)^84^, applying MOE QuickPrep (‘Delete Water Molecules Farther than 4.5 Å from Ligand or Receptor’ = True; ‘Retain QuickPrep Minimization Restraints’ = True;), and MOE minimize for energy minimization with Amber10:EHT. After the model quality inspection by Ramachandran plots, all compounds were standardized at pH of 7 prior to docking using MOE. Crystalized ligands and JAK inhibitors were docked using the software GOLD (v. 5.5)^86^ within MOE software (v.2016.08)^84^ (Efficiency = ’default’, Score Efficiency = 100; Early Termination = [number:3, RMS = 1.5]), using either GoldScore (PDB ID: 4CRS) or PLP (PDB IDs: 5C28) as scoring functions (Rigid Receptor). The poses were refined with MOE GBVI/WSA dG. For each ligand, 90 poses were generated, 15 refined and scored using the assigned scoring function.

The scoring function for each macromolecular target considered (GoldScore or PLP) was chosen based on a re-docking analysis (i.e. the scoring function minimizing the root mean square deviation [RMSD] of the crystallized ligand was selected. Re-docking of the crystallized ligand phosphothiophosphoric acid-adenylate ester in the binding site of PKN2 led to led to a RMSD value of 0.80 Å for 4CRS, while re-docking of 6-chloro-2-cyclopropyl-5-methylpyrimidin-4-amine in the binding site of PDE10A led to a value of RMSD = 0.23 Å for 5C28. JAK inhibitors were docked into the crystalized structure (baricitinib and tofacitinib using GoldScore scoring function on PKN2 [PDB ID: 4CRS], and baricitinib using PLP scoring function on PDE10A [PDB ID: 5C28]) and the minimum energy pose was chosen for the analysis.

### Statistical analysis

#### Transient receptor potential cation channel subfamily M member 6 (TRPM6)

The binding constant (*K*_d_) was calculated with a standard dose–response curve using the Hill Eq. (1):

1 $$Response=Background+\frac{Signal-Background}{1+({{K}_{d}}^{Hill\,Slope}/ {Dose}^{Hill\,Slope})}$$

The curve was fitted using a non-linear least square fit with the Levenberg–Marquardt algorithm using RStudio Team (2020) v. 1.3.1073 (RStudio, PBC, Boston, MA, http://www.rstudio.com/).

#### Protein kinase N2 (PKN2)

The IC_50_ values for baricitinib and tofacitinib were determined by a non-linear, least squares regression analysis using RStudio Team (2020) v. 1.3.1073 (RStudio, PBC, Boston, MA, http://www.rstudio.com/). Inhibitory constant (*K*i) values were estimated from experimental IC_50_ values using a web-based tool, based on the equation of Cheng and Prusoff^87^ and the observed IC_50_ of the tested compound.

#### Phosphodiesterase 10A2 (PDE10A2)

The IC_50_ value and the standard error of the mean (SEM) were determined by a non-linear, least squares regression analysis using GraphPad Prism Version 9.0.2 for Macintosh (GraphPad Software, San Diego, California USA, https://www.graphpad.com). The dose–response curve was plotted using RStudio Team (2020), v. 1.3.1073 (RStudio, PBC, Boston, MA, http://www.rstudio.com/). Inhibitory constant (*K*i) values were estimated from experimental IC_50_ values using a web-based tool, based on the equation of Cheng and Prusoff^87^ and the observed IC_50_ of the tested compound.

## Supplementary Information

Supplementary Information.

## Data Availability

All data generated or analyzed during this study are included in this published article (and its Supplementary Information files S1). Any additional materials will be provided upon request. Requests for the materials should be submitted to A.M.
