# Supplementary material for: Identification of novel off targets of baricitinib and tofacitinib by machine learning with a focus on thrombosis and viral infection
Source: Sci Rep. 2022 May 12;12:7843. doi: 10.1038/s41598-022-11879-1 (PMC9096754; doi:10.1038/s41598-022-11879-1)
Supplement: Supplementary file 1 — Supplementary Information. [file 41598_2022_11879_MOESM1_ESM.pdf]

Supplementary Information for

Identification of novel off targets of baricitinib and tofacitinib by machine learning with a focus on thrombosis and viral infection

Authors: Maria L. Faquetti, Francesca Grisoni, Petra Schneider, Gisbert Schneider, Andrea M. Burden

Supplementary Information

Fig. S1. Dose-response curve of tofacitinib on TRPM6.

Fig S2. Dose-response curve of baricitinib on PKN2.

Fig S3. Dose-response curve of tofacitinib on PKN2.

Fig S4. Dose-response curve of baricitinib on PDE10A2.

Table S1. Relevant drug-targets for baricitinib and tofacitinib predicted by Target Inference Generator (TIGER).

Table S2. Relevant drug-targets for baricitinib and tofacitinib predicted using SOM-based Prediction of Drug Equivalence Relationships (SPiDER).

Table S3. Raw in vitro results using biochemical screening assays.

Table S4. Drug-target interactions for baricitinib and tofacitinib predicted using the Similarity Ensemble Approach (SEA) and the Swiss Target Prediction.

Supplementary Information

Serine/threonine-protein Kinase N2 (PKN2) Assay

PKN2 (human) was incubated with 50 mM Tris pH 7.5, 0.1 mM EGTA, 0.1% 5-mercaptoethanol, 30 μM AKRRRLSSLRA, 10 mM magnesium acetate and [γ-33P]-ATP (15 μM). The reaction was initiated by the addition of the Mg/ATP mixture. After incubation for 40 minutes at 25 °C, the reaction was terminated by the addition of phosphoric acid (conc. 0.5%). 10 μL of the reaction was then spotted onto a P30 filtermat and washed four times for 4 minutes in 0.425% phosphoric acid and one time in methanol prior to drying and scintillation counting. Stausporine was used as reference compound. A primary screening in duplicated was carried out (30 μM) to determine the target activity. The result was expressed as a percentage of the positive control (i.e. % kinase activity remaining), and the mean of the replicates was calculated. Later, tofacitinib and baricitinib were tested at 9 concentrations (100 μM as the highest concentration and dilution factor in a half-log-scale) in duplicate for dose-response curve and IC_{50} determination. (Eurofins Cerep assay ID: 14-549KP)

Epidermal Growth Factor Receptor (EGFR) Assay

Baricitinib was combined to EGFR and incubated with 8 mM MOPS pH 7.0, 0.2 mM EDTA, 10 mM MnCl2, 0.1 mg/mL poly(Glu, Tyr) 4:1, 10 mM magnesium acetate and [γ-33P]-ATP (10 μM). The reaction was initiated by the addition of the Mg/ATP mix. After incubation for 40 minutes at 25 °C, the reaction was terminated by the addition of phosphoric acid to a concentration of 0.5%. 10 μL of the reaction was subsequently spotted onto a Filtermat A and washed four times for 4 minutes in 0.425% phosphoric acid and one time in methanol prior to drying and scintillation counting. Stausporine was used as reference compound. A primary

screening in duplicated was carried out (30 μM) to determine the target activity. The result was expressed as a percentage of the positive control (i.e. % kinase activity remaining), and the mean of the replicates was calculated. (Eurofins Cerep assay ID: 14-531KP)

Transient receptor potential cation channel subfamily M member 6 (TRPM6) Assay

HEK-293 cells were tagged with DNA for quantitative polymerase chain reaction (qPCR) detection. streptavidin-coated magnetic beads were treated with biotinylated affinity ligands for 30 minutes at 25 °C to produce affinity resins. The liganded beads were blocked with excess biotin and washed with blocking buffer (SeaBlock [Pierce], 1% BSA, 0.05% Tween 20, 1 mM DTT) to remove unbound ligand and to reduce nonspecific binding. Binding reactions were performed by combining the kinase, liganded affinity beads and tofacitinib in 1x binding buffer (20% SeaBlock, 0.17× PBS, 0.05% Tween 20, 6 mM DTT). Tofacitinib was prepared as 111x stocks in DMSO and diluted into the assay environment, such as the final concentration of DMSO was 0.9 %. Kd was determined using an 11-point 3-fold compound dilution series with three DMSO control points and DMSO was added to control assays lacking tofacitinib. Tofacitinib highest concentration was equal to 30 μM and the analysis was performed in duplicate. The analysis was carried on in polypropylene 384-well plates incubated at 25 °C with shaking for 1 h in a final volume of 20 μL. The affinity beads were washed extensively with wash buffer (1× PBS, 0.05% Tween 20) to remove unbound protein. The beads were resuspended in elution buffer (1× PBS, 0.05% Tween 20, 0.5 μM nonbiotinylated affinity ligand) and incubated at 25 °C with shaking for 30 minutes. The kinase concentration in the eluates was measured by qPCR. (Eurofins Cerep assay ID: 87-0007-1084)

Inducible NOS (iNOS) Assay

Mouse recombinant iNOS expressed in *Escherichia coli* (*E.coli*) was used to access the enzyme functional activity. Baricitinib and 1.5 U/mL of enzyme in Tris buffer pH 8.0 were combined

with 0.1 mM L-Arginine. The mixture was incubated for 90 minutes at 37 °C, followed by the addition of Griess reagent. The binding was determined by photometry at 545 nm. 1400W was used as reference compound. Baricitinib enzyme inhibition effect was calculated as a % inhibition of control enzyme activity. A primary screening in duplicated was carried out (30 µM) to determine the target activity. (Eurofins Cerep assay ID: 3185)

PI3 Kinase (p110b/p85a). The enzyme was incubated in assay buffer containing 10 µM phosphatidylinositol-4, 5-bisphosphate and Mg/ATP (200 µM). The reaction was initiated by the addition of the Mg/ATP mixture. After incubation for 30 minutes at 25 °C, the reaction was terminated by the addition of a solution containing EDTA and biotinylated phosphatidylinositol- 3,4,5-trisphosphate. Detection buffer containing europium-labelled anti-GST monoclonal antibody, GST-tagged GRP1 PH domain and streptavidin-allophycocyanin was added. Following, the plate was read in time-resolved fluorescence mode and the homogeneous time-resolved fluorescence (HTRF) signal was determined according to the formula HTRF = 10000 x (Em665 nm/Em620 nm). PI-103 was used as reference compound. A primary screening in duplicated was carried out (30 µM) to determine the target activity. The result was expressed as a percentage of the positive control (i.e. % kinase activity remaining), and the mean of the replicates was calculated. (Eurofins Cerep assay ID: 14-603KP)

Arachidonate 15-lipoxygenase (15-ALOX)

Human recombinant 15-ALOX expressed in *E. coli* cells was used. Tofacitinib and 1% DMSO were combined with 16 µg/mL of enzyme for 15 minutes at 37 °C in 50 µM Tris buffer pH 7.4. The reaction was initiated by addition of 25 µM arachidonic acid and dye DHR123 for 30 minutes at 37 °C. The binding was determined by Spectrofluorimetric quantitation of rhodamine 123 (Excitation: 485 nm, Emission: 535 nm). 2-TEDC was used as a reference compound. A primary screening in duplicated was carried out (30 µM) to determine the target

activity. Later, the analysis was repeated using 10 μM arachidonic acid and tofacitinib was tested at 8 concentrations (100 μM as the highest concentration and dilution factor in a log-scale) in triplicate for dose-response curve and IC_{50} determination. (Eurofins Cerep assay ID: 199017)

Phosphodiesterase 10A2 (PDE10A2)

The effects of baricitinib on the activity of human PDE10A2 was accessed by measuring the formation of 5'AMP from cAMP using human recombinant enzyme expressed in Sf9 cells. The test compound was added to a buffer containing 40 mM Tris/HCl (pH 7.4), 8 mM MgCl2, and 0.2 μM [3H]cAMP + cAMP. Thereafter, enzyme (about 0.8 U) was added and the mixture incubated for 20 minutes at 25 °C. SPA beads were added and the mixture was maintained at 25 °C for 30 minutes under shaking. [3H]5'AMP was quantified via scintillation counting. The results are expressed as a percent inhibition of the control enzyme activity. Papaverine was used as standard inhibitory reference compound. A primary screening in duplicated was carried out (30 μM) to determine the target activity. Later, baricitinib was tested at 8 concentrations (100 μM highest concentration and dilution factor in a log-scale) in triplicate for dose-response curve and IC_{50} determination. (Eurofins Cerep assay ID: 4080)

Adenosine A3 receptor (ADORA3)

Functional activity of ADORA3 was evaluated in CHO cells transfected with human ADORA3. Cells were incubated with tofacitinib alone or in competition with control agonist (100 nM IB-MECA) in a first assay, and in a second one with antagonist (10 nM IB-MECA) for 20 minutes at 37 °C, and formation of cAMP was determined by homogeneous time resolved fluorescence (HTRF) at λex=337 nm and λem=620 and 665 nm using a microplate reader (Envison, Perkin Elmer). The cAMP concentration was determined by dividing the

signal measured at 665 nm by that measured at 620 nm (ratio). Tofacitinib was tested at 8 concentrations (30 µM as the higher concentration and using log as dilution factor) for dose-response curve and IC_{50} determination and 8 concentrations (30 µM as the higher concentration and using log as dilution factor) for EC_{50} determination.

For the agonist mode, results were expressed as a percent of the control response to 100 nM IB-MECA, while for the antagonist mode, results were expressed as a percent inhibition of the control response to 10 nM IB-MECA. The standard reference agonist (IB-MECA) and antagonist (MRS 1220) were tested in the experiment at several concentrations to generate a concentration-response curve from which its EC_{50} and IC_{50} values, respectively, were calculated. (Eurofins Cerep assay ID: G107)

Adenosine A2A receptor (AA2AR)

Functional activity on AA2AR was evaluated in a calcium flux assay in RBL-2H3 cells expressing human AA2AR. Cell lines were expanded from freezer stocks according to standard procedures. Cells were seeded in a total volume of 20 µL into black-walled, clear-bottom, Poly-D-lysine coated 384-well microplates and incubated at 37 °C for the appropriate time prior to testing. Assays were performed in 1 x Dye Loading Buffer consisting of 1x Dye, 1x Additive A and 2.5 mM Probenecid in HBSS / 20 mM Hepes. Cells were loaded with dye prior to testing. Media was aspirated from cells and replaced with 20 µL Dye Loading Buffer. Cells were incubated for 30-60 minutes at 37 °C. Cells were pre-incubated with test sample followed by addition of agonist control (0.045 µM NECA). Intermediate dilution of sample stocks was performed to generate 3x sample in assay buffer. After dye loading, cells were removed from the incubator and 10 µL 3x sample was added. Cells were incubated at 25 ºC over 30 minutes in the dark to equilibrate plate temperature. Compound activity was measured on a FLIPR Tetra (MDS). Calcium mobilization was monitored for 2 minutes and 10 µM agonist control in HBSS

/ 20 mM Hepes was added to the cells 5 seconds into the assay. Baricitinib and Tofacitinib were tested at 10 concentrations (30 μM as the higher concentration and using log as dilution factor) for dose-response curve and IC_{50} determination. Cellular antagonist effect was calculated as a % inhibition of control reference agonist response. (Eurofins Cerep assay ID: 86-0011P-2409AN).

Fig S1. Dose-response curve of tofacitinib on TRPM6. The amount of kinase measured by qPCR (Signal; y-axis) is plotted against the corresponding compound concentration in nM in log10 scale (x-axis) per replicate (n=2).

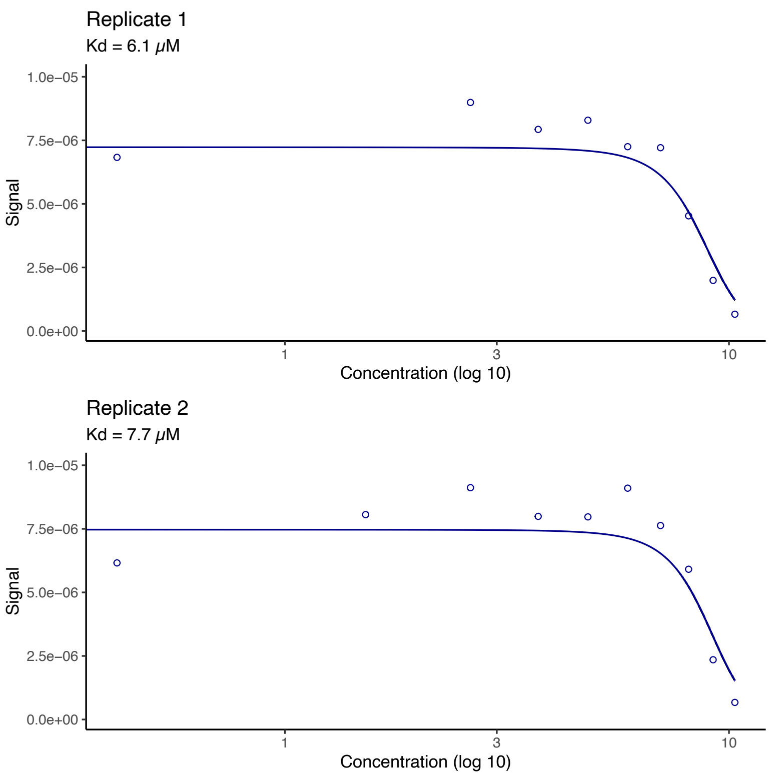

Fig S2. Dose-response curve of baricitinib on PKN2. The activity measured (% of control; y-axis) is plotted against the corresponding compound concentration in M in half-log10 scale (x-axis) per replicate (n=2).

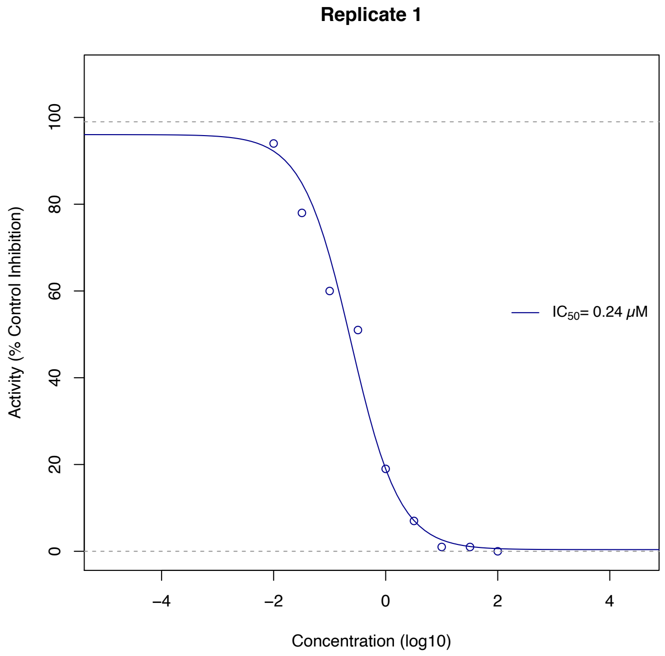

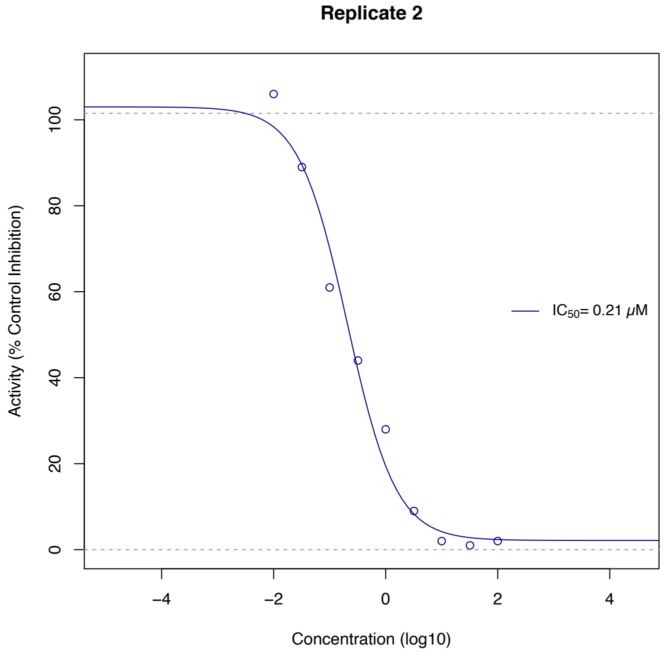

Fig S3. Dose-response curve of tofacitinib on PKN2. The activity measured (% of control; y-axis) is plotted against the corresponding compound concentration in M in half-log10 scale (x-axis) per replicate (n=2).

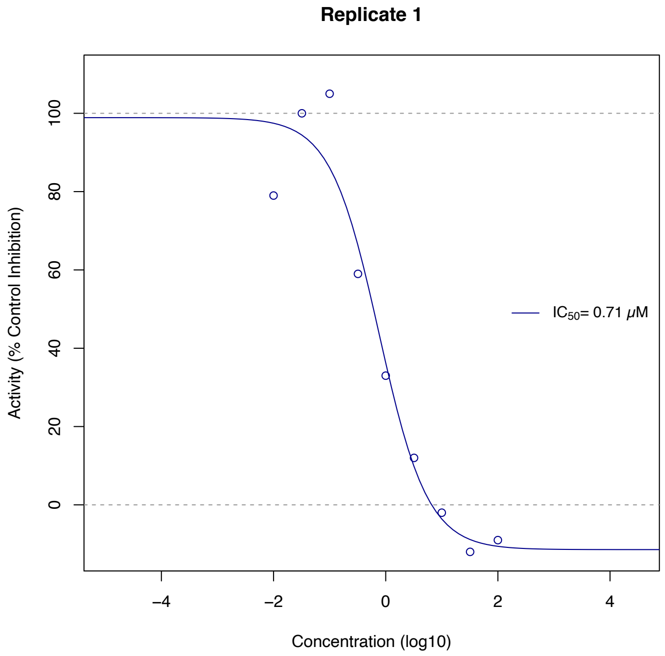

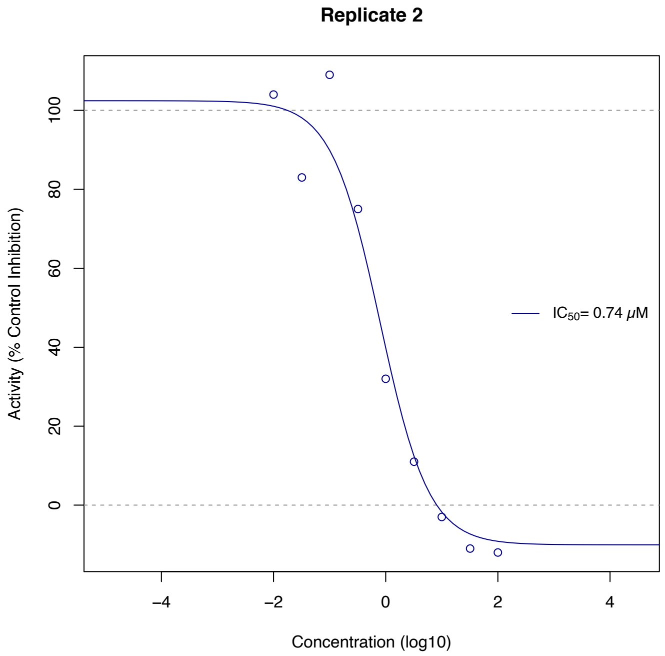

Fig S4. Dose-response curve of baricitinib on PDE10A2. The activity measured (% of inhibition of control enzyme activity; y-axis) is plotted against the corresponding compound concentration in M in log10 scale (x-axis).

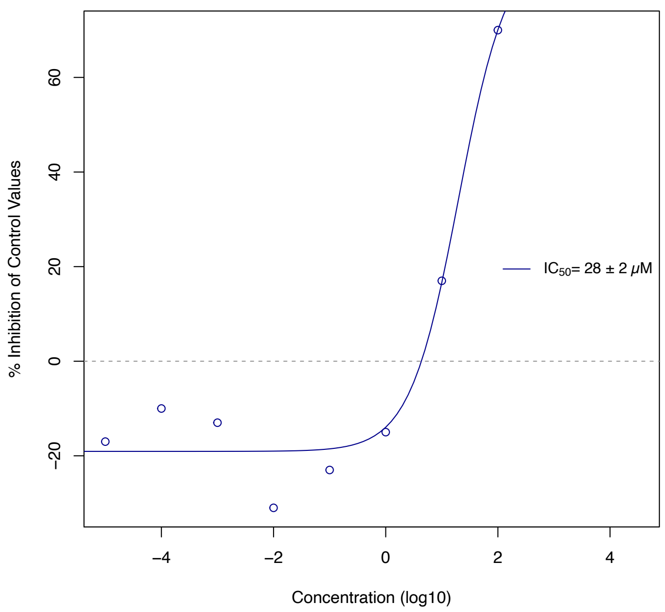

Table S1. Relevant drug-target interactions for baricitinib and tofacitinib predicted using the Target Inference Generator (TIGER). Targets with statistically meaningful predictions from TIGER (score >1) were identified.

Drug	Prediction	TIGER Score
Baricitinib	Deoxycytidine Kinase inhibitor	8.8
	Janus Kinase 3 Inhibitor	8.4
	Phosphodiesterase 6A Inhibitor	7.9
	Muscarinic Acetylcholine Receptor 4 Allosteric Modulator	6.8
	Protein Kinase C Beta Inhibitor	6.8
	DNA Gyrase subunit B (GyrB) Inhibitor (*Mycobacterium smegmatis*)	6
	Metabotropic Glutamate Receptor 1 Antagonist	6
	Leucine-Rich Repeat Serine-Threonine-Protein Kinase 2 Inhibitor	6
	Mitogen-Activated Protein Kinase Kinase Kinase 12 Inhibitor	5.3
	Adenosine Receptor A2A Inhibitor	4.5
	Epidermal Growth Factor Receptor Tyrosine Kinase 1 Inhibitor	4.2
	Leucine-Rich Repeat Serine-Threonine-Protein Kinase 2 Allosteric Modulator	4.1
	Calcium/Calmodulin Dependent Protein Kinase IV CaMK4 Inhibitor	3.8
	Inducible Nitric Oxide Synthase InOS Inhibitor	3.3
	Carbonic Anhydrase I Inhibitor	3
	Testis Specific Serine Kinase 2 Inhibitor	2.9
	Serine Threonine Kinase ILK1 (p59ILK, Integrin-Linked Kinase) Inhibitor	2.9
	MAP Kinase Interacting Serine Threonine Protein Kinase 1 Mnk Allosteric Modulator	2.8
	Methionine Aminopeptidase Inhibitor	2.8
	GPR119 (KIF19, AXOR103, G-Protein Coupled Receptor PGR2) Agonist	2.7
	Ribosomal Protein S6 Kinase A1 RSK1 (p90RSK) Inhibitor	2.4
	Phosphodiesterase 10A Inhibitor	2.1

	Methionine Aminopeptidase 1 Inhibitor	1.9
	Casein Kinase 2, Beta Polypeptide Subunit CK2-B Inhibitor	1.8
	Polo-Like Kinase 4 (Serine Threonine Kinase Sak, STK18) Inhibitor	1.8
	Carbonic Anhydrase II Inhibitor	1.7
	PI3-Kinase P110-Alpha Subunit Inhibitor	1.7
	Ras-Related Protein Rab-7a Inhibitor	1.4
	Carbonic Anhydrase IX Inhibitor	1.3
	GPR39 Agonist	1.2
	Thymidine Kinase (HSV) Inhibitor	1.1
**Tofacitinib**	D-Amino-Acid Oxidase Inhibitor	11.3
	Phosphodiesterase 8B Inhibitor	10.8
	Metabotropic Glutamate Receptor 5 Inhibitor	7.3
	Janus Kinase 3 Inhibitor	6.9
	M18 Aspartyl Aminopeptidase Inhibitor	6.2
	Tyrosine-Protein Kinase TYK2 Inhibitor	5.3
	6-O-Methylguanine-DNA Methyltransferase Inhibitor	4.6
	Arachidonate 15-Lipoxygenase 15-ALOX Inhibitor	4.4
	Histone Lysine N-Methyltransferase EMTH1 (H3 Lysine-9 Specific 5) Inhibitor	4.3
	Histone Lysine N-Methyltransferase EHMT2 (Euchromatic Histone Methyltransferase 2, G9a) Inhibitor	3.8
	Ulp1 Endopeptidase, SuMO Specific Peptidase, Sentrin-Specific Protease 7 Inhibitor	3.8
	Glutaminyl Cyclase GC Inhibitor	3.1
	Ubiquitin-Conjugating Enzyme E2 N Inhibitor	3.1
	Transketolase Inhibitor	2.9
	Methionine Aminopeptidase 1 Inhibitor	2.8
	Leucine-Rich Repeat Serine-Threonine-Protein Kinase 2 Allosteric Modulator	2.7
	Hypoxia-Inducible Factor Prolyl Hydroxylase 1 (egl-9 Family Hypoxia Inducible Factor 2) Inhibitor	2.6

Adenosine Receptor A2A Antagonist	2.5
TRP Canonical Subfamily M Member 6 Inhibitor	2.3
Ulp1 Endopeptidase. SuMO Specific Peptidase. Sentrin-Specific Protease 6 Inhibitor	2.3
TRP Canonical Subfamily M Member 3 Inhibitor	2.2
Methionine Aminopeptidase Inhibitor	2.1
Lysine-Specific Demethylase 4C (JHDM3C, Jumonj Domain Containing 2C) Inhibitor	2
Doublecortin Like Kinase 3 Inhibitor	2
Serine Threonine Kinase ILK1 (p59ILK, Integrin-Linked Kinase) Inhibitor	2
Janus Kinase 1 Inhibitor	1.9
Ulp1 Endopeptidase, SuMO Specific Peptidase, Sentrin-Specific Protease 8 Inhibitor	1.9
Ribosomal Protein S6 Kinase A6 Inhibitor	1.8
Serine Threonine Kinase 4 Inhibitor	1.8
Ectonucleotide Pyrophosphatase/phosphodiesterase Family Member 1 Inhibitor	1.7
Non-Receptor Tyrosine-Protein Kinase TNK1 Inhibitor	1.7
Mitogen-Activated Protein Kinase Kinase Kinase Kinase 3 Inhibitor	1.7
11Beta-Hydroxysteroid Dehydrogenase Inhibitor	1.6
Protein Kinase N 1 Inhibitor	1.6
Protein Kinase N 2 Inhibitor	1.6
Microtubule Affinity Regulating Kinase 4 Inhibitor	1.6
NUAK Family SNF1-Like Kinase 2 Inhibitor	1.6
Exportin-1 Inhibitor	1.6
Glutathione S-Transferase GST P1-1 Inhibitor	1.4
Glutathione S-Transferase Mu 2 Inhibitor	1.4
Muscarinic Acetylcholine Receptor 5 Inhibitor	1.4
Inducible Nitric Oxide Synthase Inhibitor	1.2
NAD-Dependent Deacetylase Sirtuin 3 Inhibitor	1.2
GPR6 Antagonist	1.2

Dihydrofolate Reductase Inhibitor	1.1
Adenosine Receptor A3 Antagonist	1.1
CDGSH iron-Sulfur Domain-Containing Protein 1 Inhibitor	1.1

Note: In the manuscript, we refer as the number of predicted targets as the number of different predictions. For TIGER, the same target can be predicted multiple times with different interaction mode (i.e. agonist, inhibitor, allosteric modulator and antagonist).

Table S2. Relevant drug-targets for baricitinib and tofacitinib predicted using SOM-based Prediction of Drug Equivalence Relationships (SPiDER). Targets with statistically meaningful predictions from SPiDER ($p < 0.05$) were identified.

Drug	Target	P-value
**Baricitinib**	Monoamine Oxidase	0.02
	Tyrosine Kinase	0.02
	Sodium Channel	0.02
	Transient Receptor Potential Ion Channel TRP	0.03
	Phosphodiesterase (3',5'-Cyclic-Nucleotide Phosphodiesterase)	0.03
	Metabotropic Glutamate Receptor	0.04
	Glucagon-Like Peptide Receptor	0.04
	Serine Threonine Kinase	0.04
	Potassium Channel	0.05
**Tofacitinib**	Phosphodiesterase (3',5'-Cyclic-Nucleotide Phosphodiesterase)	0.01
	Tyrosine Kinase	0.01
	Serine Threonine Kinase	0.02
	Metabotropic Glutamate Receptor	0.02
	Sodium Channel	0.02
	Endopeptidase (Cysteine Endopeptidase, Cysteine Protease)	0.02
	Potassium Channel	0.04
	Exopeptidase (Serine Exopeptidase, Serine Protease)	0.04
	Histamin Receptor	0.04
	Muscarinic Acetylcholine Receptor	0.05
	Androgen Receptor	0.05

Table S3. Raw in vitro results using biochemical screening assays. The experimental tests were performed at 30 µM and technical replicates (n=2).

Assay	Study ID	Eurofins Assay ID	Drug	% inhibition of control activity (1st)	% inhibition of control activity (2nd)	% inhibition (mean) activity	Follow-up experiment
PRK2 Human AGC Kinase Enzymatic Radiometric Assay	FR095-0022152	14-549KP	baricitinib	1[a]	2[a]	98%	yes
PI3Kbeta (PIK3CB) (p110b/p85a) Human PtdIns(4,5)P 3-Kinase Enzymatic HTRF Assay	FR095-0020731	14-603KP	baricitinib	92[a]	99[a]	4%	no
EGFR Human RTK Kinase Enzymatic Radiometric Assay	FR095-0020731	14-531KP	baricitinib	81[a]	77[a]	21%	no
iNOS Mouse Nitric Oxide Synthase Enzymatic Assay	FR095-0020731	3185	baricitinib	6.0	4.0	5%	no
PDE10A2 Human Phosphodiesterase Enzymatic Assay	FR095-0020731	4080	baricitinib	45.5	44.0	44.8%	yes

PRK2 Human AGC Kinase Enzymatic Radiometric Assay	FR095-0020731	14-549KP	tofacitinib	3[a]	0[a]	98%	yes
Transient Receptor Potential Cation Channel Subfamily M member 6 (TRPM6)	US073-0013667	87-0007-1084	tofacitinib	6.1[b]	5.7[b]	n.d.	no
15-LOX-1 Human Lipoxygenase Enzymatic Assay	FR095-0020731	199017	tofacitinib	19.3	34.0	27%	yes

[a] measured as % of positive control activity

[b] activity measured as K_d (μM)

Table S4. Drug-target interactions for baricitinib and tofacitinib predicted using the Similarity Ensemble Approach (SEA) and the Swiss TargetPrediction.

	Prediction tool				
Drug	SEA[a]	Z-score	p-value	Swiss Target Prediction (Probability)[b]	Probability
Baricitinib	AP2-associated protein kinase 1	8.6174	8.9e-06	Tyrosine-protein kinase JAK3	1.0
	Ankyrin repeat and protein kinase domain-containing protein 1	13.7999	1,155e-05	Tyrosine-protein kinase JAK1	1.0
	BMP-2-inducible protein kinase	18.2298	3,938e-08	Tyrosine-protein kinase JAK2	1.0
	Bone morphogenetic protein receptor type-2	12.6351	5,146e-05	JAK3/JAK1	1.0
	Bone morphogenetic protein receptor type-1B	9.4168	3,192e-03	JAK1/JAK2/TYK2	1.0
	Peripheral plasma membrane protein CASK	20.8241	1,413e-09	JAK1/TYK2	1.0
	Death-associated protein kinase 1	10.4079	8,955e-04	JAK2/TYK2	1.0
	Death-associated protein kinase 2	10.8760	4,913e-04	Tyrosine-protein kinase TYK2	1.0
	Serine/threonine-protein kinase DCLK1	10.3159	1,008e-03	CDC7/DBF4 (Cell division cycle 7-related protein kinase/Activator of S phase kinase)	0.11
	Serine/threonine-protein kinase DCLK2	19.7327	5,729e-09	Leucine-rich repeat serine/threonine-protein kinase 2	0.11
	Serine/threonine-protein kinase DCLK3	20.0268	3,929e-09	Rho-associated protein kinase 1	0.11
	Myotonin-protein kinase	21.0990	9,933e-10	Neuropeptide Y receptor type 5	0.11
	Ephrin type-B receptor 6	8.6174	8.9e-06	ALK tyrosine kinase receptor	0.11
	Rhodopsin kinase GRK1	12.2963	7,948e-05	Beta-adrenergic receptor kinase 2	0.11

G protein-coupled receptor kinase 4	12.2963	7,948e-05	G-protein coupled receptor kinase 2	0.11
G protein-coupled receptor kinase 5	8.7348	7,656e-03	Orexin receptor 2	0.11
Rhodopsin kinase GRK7	19.0775	1,328e-08	c-Jun N-terminal kinase 1	0.11
Homeodomain-interacting protein kinase 1	9.0776	4,932e-03	Lysine-specific demethylase 4C	0.11
Homeodomain-interacting protein kinase 3	10.1908	1,183e-03	Adenosine A1 receptor	0.11
Tyrosine-protein kinase JAK2	95.2524	4,935e-51	Adenosine A2a receptor	0.11
Tyrosine-protein kinase JAK1	119.2066	2,243e-64	Adenosine A2b receptor	0.11
Tyrosine-protein kinase JAK2	21.2133	8,579e-10	Adenosine A3 receptor	0.11
Tyrosine-protein kinase JAK3	17.4948	1,011e-07	Cyclin-dependent kinase 2	0.11
Tyrosine-protein kinase JAK3	69.2252	1,551e-36	Glutaminyl-peptide cyclotransferase	0.11
Tyrosine-protein kinase JAK3	111.8245	2,901e-60	Prokineticin receptor 1	0.11
Calcium/calmodulin-dependent protein kinase type 1G	16.4586	3,818e-07	Nerve growth factor receptor Trk-A	0.11
Calcium/calmodulin-dependent protein kinase type 1D	17.4635	1,052e-07	Neurotrophic tyrosine kinase receptor type 2	0.11
Calcium/calmodulin-dependent protein kinase type II subunit α	12.8031	4,149e-05	NT-3 growth factor receptor	0.11
Ribosomal protein S6 kinase α-6	18.2298	3,938e-08	Cytochrome P450 19A1	0.11

Mitogen-activated protein kinase kinase kinase 15	25.2463	4,885e-12	Tyrosine-protein kinase ITK/TSK	0.11
Mitogen-activated protein kinase kinase kinase 1	17.9162	5,887e-08	cAMP-dependent protein kinase alpha-catalytic subunit	0.11
Mitogen-activated protein kinase kinase kinase 2	10.1908	1,183e-03	Phosphodiesterase 10A	0.11
Mitogen-activated protein kinase kinase kinase 3	11.3960	2,522e-04	Corticotropin releasing factor receptor 1	0.11
Microtubule-associated serine/threonine-protein kinase 1	20.8241	1,413e-09	Cyclin-dependent kinase 2/cyclin A	0.11
Dual specificity mitogen-activated protein kinase kinase 2	9.2210	4,104e-03	Poly [ADP-ribose] polymerase-1	0.11
Dual specificity mitogen-activated protein kinase kinase 3	11.6786	1,755e-04	Metabotropic glutamate receptor 5	0.11
Dual specificity mitogen-activated protein kinase kinase 4	11.3960	2,522e-04	MAP kinase signal-integrating kinase 2	0.11
Serine/threonine-protein kinase Nek3	29.8431	1,338e-14	Interleukin-8 receptor B	0.11
Serine/threonine-protein kinase Nek7	18.7712	1,967e-08	Signal transducer and activator of transcription 3	0.11

NUAK family SNF1-like kinase 2	17.4673	1,047e-07	CDC7/DBF4 (Cell division cycle 7-related protein kinase/Activator of S phase kinase)	0.11
Serine/threonine-protein kinase OSR1	20.8241	1,413e-09	Glycogen synthase kinase-3 beta	0.11
Serine/threonine-protein kinase PAK 5	13.7999	1,155e-05	Focal adhesion kinase 1	0.11
Serine/threonine-protein kinase PAK 6	15.8308	8.54e-10	HERG	0.11
Phosphorylase b kinase gamma catalytic chain, skeletal muscle/heart isoform	11.6786	1,755e-04	Tyrosine-protein kinase ABL	0.11
Serine/threonine-protein kinase PknB	10.3159	1,008e-03	Anandamide amidohydrolase	0.11
Serine/threonine-protein kinase RIO1	12.9969	3,236e-05	Carbonic anhydrase I	0.11
Serine/threonine-protein kinase RIO2	12.9969	3,236e-05	Carbonic anhydrase IX	0.11
Serine/threonine-protein kinase RIO3	12.9969	3,236e-05	Myosin light chain kinase, smooth muscle	0.11
Receptor-interacting serine/threonine-protein kinase 4	15.8308	8.54e-10	Casein kinase II alpha	0.11
Serine/threonine-protein kinase SBK1	16.4586	3,818e-07	Glycogen synthase kinase-3 alpha	0.11
Uncharacterized serine/threonine-protein kinase SBK3	18.7712	1,967e-08	Histone acetyltransferase p300	0.11
Serine/threonine-protein kinase SIK3	18.7712	1,967e-08	Orexin receptor 1	0.11
SRSF protein kinase 3	12.6351	5,146e-05	Dual-specificity tyrosine-phosphorylation regulated kinase 1A	0.11
Serine/threonine-protein kinase 16	10.6360	6,684e-04	Dual specificity mitogen-activated protein kinase kinase 1	0.11

Serine/threonine-protein kinase 25	13.3841	1,969e-05	Epidermal growth factor receptor erbB1	0.11
Serine/threonine-protein kinase 26	10.1908	1,183e-03	Serine/threonine-protein kinase PIM1	0.11
STE20/SPS1-related proline-alanine-rich protein kinase	20.8241	1,413e-09	Serine/threonine-protein kinase PIM2	0.11
Threonine--tRNA ligase 1, cytoplasmic	21.3575	7.13e-13	Lysine-specific demethylase 4D-like	0.11
Threonine--tRNA ligase	21.3575	7.13e-13	Cyclin-dependent kinase 5/CDK5 activator 1	0.11
Serine/threonine-protein kinase TAO2	10.8760	4,913e-04	Cyclin-dependent kinase 4/cyclin D1	0.11
Serine/threonine-protein kinase TNNI3K	30.4958	5,794e-15	Cyclin-dependent kinase 2/cyclin E	0.11
Targeting protein for Xklp2	18.6374	2,335e-08	Cyclin-dependent kinase 1/cyclin B	0.11
Non-receptor tyrosine-protein kinase TYK2	85.7151	1,013e-45	Lysine-specific demethylase 5C	0.11
Serine/threonine-protein kinase ULK1	11.9781	1,195e-04	Casein kinase I delta	0.11
Serine/threonine-protein kinase ULK2	11.6786	1,755e-04	Protein kinase C gamma (by homology)	0.11
Serine/threonine-protein kinase ULK3	21.6896	4,657e-10	Cytochrome P450 17A1	0.11
Serine/threonine-protein kinase VRK2	37.2957	9.45e-22	Lysine-specific demethylase 4A	0.11
			Lysine-specific demethylase 4D	0.11
			Neprilysin (by homology)	0.11
			Phosphodiesterase 5A	0.11
			Melatonin receptor 1A	0.11
			GABA-A receptor; alpha-1/beta-3/gamma-2	0.11

	Acyl coenzyme A:cholesterol acyltransferase	0.11
	G protein-coupled receptor kinase 7	0.11
	Serine/threonine-protein kinase TAO2	0.11
	Serine/threonine-protein kinase/endoribonuclease IRE1	0.11
	Serine/threonine-protein kinase OSR1	0.11
	STE20/SPS1-related proline-alanine-rich protein kinase	0.11
	Mitogen-activated protein kinase kinase kinase 15	0.11
	Microtubule-associated serine/threonine-protein kinase 1	0.11
	Serine/threonine-protein kinase SBK1	0.11
	Serine/threonine-protein kinase VRK2	0.11
	Peripheral plasma membrane protein CASK	0.11
	Stem cell growth factor receptor	0.11
	Inhibitor of nuclear factor kappa B kinase beta subunit	0.11
	Kinesin-1 heavy chain/ Tyrosine-protein kinase receptor RET	0.11
	Dual specificity mitogen-activated protein kinase kinase 3	0.11
	Phosphorylase kinase gamma subunit 2	0.11
	Death-associated protein kinase 3	0.11
	CaM kinase I alpha	0.11
	Ribosomal protein S6 kinase alpha 1	0.11

				Death-associated protein kinase 1	0.11
				Casein kinase I alpha	0.11
				CaM kinase II	0.11
				cGMP-dependent protein kinase 2	0.11
				Dual specificity mitogen-activated protein kinase kinase 4	0.11
				Dual specificity mitogen-activated protein kinase kinase 2	0.11
				Rho-associated protein kinase 2	0.11
				Serine/threonine-protein kinase PLK1	0.11
				JAK2/JAK1	0.11
**Tofacitinib**	Tyrosine-protein kinase ABL1	0.1191	0.3824	Tyrosine-protein kinase JAK3	0.78
	RAC-beta serine/threonine-protein kinase	43.2978	4,288e-22	Tyrosine-protein kinase JAK2	0.78
	RAC-gamma serine/threonine-protein kinase	42.6288	1,011e-21	Mitogen-activated protein kinase kinase kinase kinase 5	0.78
	RAC-alpha serine/threonine-protein kinase	16.2343	5.09e-10	Mitogen-activated protein kinase kinase kinase kinase 3	0.78
	Ankyrin repeat and protein kinase domain-containing protein 1	9.1999	4,216e-03	Tyrosine-protein kinase JAK1	0.67
	BMP-2-inducible protein kinase	30.6752	4,603e-15	Serine/threonine-protein kinase MST1	0.66
	Bromodomain-containing protein 9	15.7274	9,752e-07	Serine/threonine-protein kinase MST2	0.66
	C-C chemokine receptor type 10	25.6727	2,776e-12	Leucine-rich repeat serine/threonine-protein kinase 2	0.27
	C-C chemokine receptor type 10	36.1292	4,219e-18	G protein-coupled receptor kinase 7	0.27

Cyclin-dependent kinase 8	4.6246	0.00149	Tyrosine-protein kinase FYN	0.27
Cat eye syndrome critical region protein 2	43.2048	4,831e-22	Tyrosine-protein kinase ABL	0.27
Peripheral plasma membrane protein CASK	13.9880	9,077e-06	Kinesin-1 heavy chain/ Tyrosine-protein kinase receptor RET	0.27
Serine/threonine-protein kinase DCLK1	17.6241	8,563e-08	CaM kinase I alpha	0.27
Serine/threonine-protein kinase DCLK2	13.2451	2,354e-05	Ribosomal protein S6 kinase alpha 1	0.27
Serine/threonine-protein kinase DCLK3	33.6483	1,016e-16	Tyrosine-protein kinase LCK	0.27
Myotonin-protein kinase	35.4233	1,043e-17	CaM kinase II	0.27
Tyrosine-protein kinase Fyn	6.9580	7,476e-02	Rho-associated protein kinase 2	0.27
Rhodopsin kinase GRK7	32.0774	7,622e-16	Protein kinase C delta	0.27
Tyrosine-protein kinase JAK1	193.7198	7,023e-106	Protein kinase N2	0.27
Tyrosine-protein kinase JAK2	159.1896	1,202e-86	JAK3/JAK1	0.27
Tyrosine-protein kinase JAK2	68.3726	4,629e-36	JAK2/JAK1	0.27
Tyrosine-protein kinase JAK3	56.5706	1,735e-29	Rho-associated protein kinase 1	0.27
Tyrosine-protein kinase JAK3	221.3393	2.9e-124	JAK1/JAK2/TYK2	0.27
Tyrosine-protein kinase JAK3	229.5137	8,113e-126	JAK1/TYK2	0.27
Calcium/calmodulin-dependent protein kinase type 1	14.5576	4,372e-06	JAK2/TYK2	0.27
Calcium/calmodulin-dependent protein kinase type 1D	21.3734	6,986e-10	Protein kinase N1	0.27

Calcium/calmodulin-dependent protein kinase type 1G	11.0143	4,114e-04	Tyrosine-protein kinase TYK2	0.27
Calcium/calmodulin-dependent protein kinase type II subunit alpha	22.3903	1,896e-10	Ribosomal protein S6 kinase alpha 2	0.27
Calcium/calmodulin-dependent protein kinase type II subunit delta	6.8500	8,586e-02	CaM kinase II alpha	0.27
Protein kinase C delta type	11.3562	2,654e-04	MAP kinase signal-integrating kinase 2	0.27
Protein kinase C gamma type	6.8956	8,099e-02	BMP-2-inducible protein kinase	0.27
Protein kinase C theta type	9.4697	2,983e-03	Ribosomal protein S6 kinase alpha 6	0.27
Ribosomal protein S6 kinase alpha-1	13.8207	1,125e-05	Serine/threonine-protein kinase ULK3	0.27
Ribosomal protein S6 kinase alpha-2	5.9388	0.0002763	CaM kinase I delta	0.27
Ribosomal protein S6 kinase alpha-6	30.6752	4,603e-15	Myotonin-protein kinase	0.27
Tyrosine-protein kinase Lck	2.0603	0.03918	Mitogen-activated protein kinase kinase kinase kinase 2	0.27
Leucine-rich repeat serine/threonine-protein kinase 2	3.9951	0.003337	Non-receptor tyrosine-protein kinase TNK1	0.27
Leukocyte tyrosine kinase receptor	9.5348	2,744e-03	Serine/threonine-protein kinase DCLK1	0.27
Mitogen-activated protein kinase kinase kinase 15	16.9960	1,916e-07	NUAK family SNF1-like kinase 2	0.27

Mitogen-activated protein kinase kinase kinase 1	12.0078	1,151e-04	Serine/threonine-protein kinase DCLK3	0.27
Mitogen-activated protein kinase kinase kinase kinase 3	21.9976	3,137e-10	Serine/threonine-protein kinase AKT2	0.10
Mitogen-activated protein kinase kinase kinase kinase 2	4.0298	0.003192	Poly [ADP-ribose] polymerase-1	0.10
Mitogen-activated protein kinase kinase kinase kinase 5	4.6443	0.001452	Adrenergic receptor alpha-2	0.10
Serine/threonine-protein kinase MARK2	8.4926	1,045e-02	Alpha-2b adrenergic receptor	0.10
MAP/microtubule affinity-regulating kinase 3	3.9001	0.003768	Dual specificity mitogen-activated protein kinase kinase 7	0.10
MAP/microtubule affinity-regulating kinase 4	21.1363	9,469e-10	Dopamine transporter (by homology)	0.10
Microtubule-associated serine/threonine-protein kinase 1	13.9880	9,077e-06	Thrombin and coagulation factor X	0.10
Mitogen-activated protein kinase 8	9.9263	1,661e-03	Dipeptidyl peptidase IV	0.10
MAP kinase-interacting serine/threonine-protein kinase 2	2.7415	0.01655	Thrombin	0.10
Serine/threonine-protein kinase Nek3	20.1200	3,487E-09	Vanilloid receptor	0.10
Serine/threonine-protein kinase Nek7	12.5902	5,451E-05	Beta-secretase 1	0.10
NUAK family SNF1-like kinase 2	29.4144	2,319E-14	Plasma kallikrein	0.10

Serine/threonine-protein kinase OSR1	13.9880	9,077E-06	Serine/threonine-protein kinase AKT	0.10
Serine/threonine-protein kinase PAK 5	9.1999	4,216E-03	Kinesin-like protein 1	0.10
Serine/threonine-protein kinase PAK 6	10.5862	7,125E-04	Beta secretase 2	0.10
Serine/threonine-protein kinase N1	20.3522	2,589E-09	Dopamine D1 receptor	0.10
Serine/threonine-protein kinase N2	12.7536	4,421E-05	Glutaminyl-peptide cyclotransferase-like protein	0.10
Serine/threonine-protein kinase PknB	17.6241	8,563E-08	Serine/threonine-protein kinase AKT	0.0
Cyclin-dependent kinase 4	8.8598	6,522E-03	Protein kinase C (PKC)	0.0
Proto-oncogene tyrosine-protein kinase receptor Ret	5.5367	0.0004626	MAP kinase ERK1	0.0
Serine/threonine-protein kinase RIO1	8.6512	8,523E-03	Voltage-gated potassium channel subunit Kv1.5	0.0
Serine/threonine-protein kinase RIO2	8.6512	8,523E-03	Voltage-gated potassium channel subunit Kv1.3	0.0
Serine/threonine-protein kinase RIO3	8.6512	8,523E-03	C-X-C chemokine receptor type 3	0.0
Receptor-interacting serine/threonine-protein kinase 4	10.5862	7,125E-04	Histamine H4 receptor	0.0
Rho-associated protein kinase 1	5.4818	0.0004964	Arachidonate 5-lipoxygenase	0.0
Rho-associated protein kinase 2	17.4908	1,016E-07	Heat shock protein HSP 90-alpha	0.0
Serine/threonine-protein kinase SBK1	11.0143	4,114E-04	Receptor protein-tyrosine kinase erbB-2	0.0
Uncharacterized serine/threonine-protein kinase SBK3	12.5902	5,451E-05	Death-associated protein kinase 3	0.0

Serine/threonine-protein kinase SIK2	17.8016	6.82e-11	Death-associated protein kinase 1	0.0
Serine/threonine-protein kinase SIK3	12.5902	5,451E-05	Death-associated protein kinase 2	0.0
STE20-like serine/threonine-protein kinase	5.5284	0.0004676	Serine/threonine-protein kinase 17B	0.0
Serine/threonine-protein kinase 25	8.9159	6,069E-03	Serine/threonine-protein kinase 17A	0.0
STE20/SPS1-related proline-alanine-rich protein kinase	13.9880	9,077E-06	Proline-rich AKT1 substrate 1	0.0
Serine/threonine-protein kinase 3	9.5702	2,622E-03	Muscarinic acetylcholine receptor M5	0.0
Serine/threonine-protein kinase 4	24.5822	1,144E-11	Melanocortin receptor 4	0.0
Transcription initiation factor TFIID subunit 1	35.9930	5,024E-18	Glutaminyl-peptide cyclotransferase	0.0
Serine/threonine-protein kinase TNNI3K	15.0347	2,371E-06	MAP kinase ERK2	0.0
Non-receptor tyrosine-protein kinase TNK1	24.5822	1,144E-11	Trace amine-associated receptor 1 (by homology)	0.0
Non-receptor tyrosine-protein kinase TYK2	152.3577	7,681E-83	Bile acid receptor FXR	0.0
Serine/threonine-protein kinase ULK3	36.4012	2,976E-18	Interleukin-1 receptor-associated kinase 4	0.0
Serine/threonine-protein kinase VRK2	25.1809	5,329E-12	Estradiol 17-beta-dehydrogenase 1	0.0
			Gamma-secretase	0.0
			Ephrin receptor	0.0
			Heat shock protein HSP 90-beta	0.0
			Serine/threonine-protein kinase RIPK2	0.0

	Nischarin	0.0
	P2X purinoceptor 7	0.0
	Cytochrome P450 19A1	0.0
	Serotonin 1d (5-HT1d) receptor	0.0
	Cyclooxygenase-1	0.0
	Sodium/hydrogen exchanger 1	0.0
	Serotonin 5a (5-HT5a) receptor	0.0
	Cyclin-dependent kinase 5/CDK5 activator 1	0.0
	Estrogen receptor alpha	0.0
	Trypsin I	0.0
	Dual-specificity tyrosine-phosphorylation regulated kinase 1A	0.0
	Estrogen receptor beta	0.0
	Serine/threonine-protein kinase Nek1	0.0
	Dopamine D2 receptor	0.0
	Voltage-gated L-type calcium channel alpha-1C subunit	0.0

[a]SEA, molecular descriptor: ECFP4 fingerprints; reference database: ChEMBL 16 binding data (activity <10 μM).

[b] Swiss Target Prediction, FP2 fingerprints; reference database: ChEMBL23 binding data (activity <10 μM).
